# The Quality of Life of Patients Living with a Urinary Catheter and Its Associated Factors: A Cross-Sectional Study in Egypt

**DOI:** 10.3390/healthcare11162266

**Published:** 2023-08-11

**Authors:** Naglaa Youssef, Ashley Shepherd, Catherine Best, Suzanne Hagen, William Mackay, Debbie Waddell, Hanan El Sebaee

**Affiliations:** 1 Department of Medical-Surgical Nursing, College of Nursing, Princess Nourah bint Abdulrahman University, P.O. Box 84428, Riyadh 11671, Saudi Arabia; 2 Faculty of Health Sciences and Sport, University of Stirling, Stirling FK9 4LA, UK; 3 Nursing, Midwifery and Allied Health Professions Research Unit, Glasgow Caledonian University, Glasgow G4 0BA, UK; 4 School of Health and Life Sciences, University of the West of Scotland, Paisley PA1 2BE, UK; 5 Medical-Surgical Nursing Department, Faculty of Nursing, Cairo University, Cairo 11562, Egypt

**Keywords:** ICIQ-LTCQoL, incontinence, indwelling catheter, quality of life, urinary catheter

## Abstract

Background: In Arabic countries, no research has focused on the experience of patients with indwelling urinary catheters. This cross-sectional study is the first to evaluate the catheter-specific quality of life (QoL) of patients living with a urinary catheter in Egypt. Methods: This study was conducted from April to September 2017, using a convenience sample of patients from a University Hospital. Data were collected using the International Consultation on Incontinence Questionnaire-Long-Term Catheter QoL (ICIQ-LTCQoL) instrument, along with a demographic datasheet. Results: 141 were enrolled, with 47.5% inpatients, 52.5% outpatients. A total of 70.9% reported problems with catheter function, and 92.2% reported that the catheter affected their daily lives. Place (inpatient or outpatient) was significantly associated with the total score of the ICIQ-LTCQoL (mean difference (MD) 6.34 (95% CI: 3.0 to 9.73)) and both subscales (catheter function subscale: MD = 4.92 (95% CI: 2.12 to 7.73) and lifestyle impact subscale: MD = 1.44 (95% CI: 0.3 to 2.63)), suggesting that outpatients have poorer QoL than inpatients. Moreover, catheter material was significantly related to the catheter function domain with Silicone Foley Catheter (100% Silicon) users experiencing poorer QoL related to catheter function than those with Latex Foley Catheter (Silicon-coated) (MD 4.43 (95% CI: 0.62 to 8.24). Workers/employees were found to have poorer QoL than those who were retired (MD = 4.94 (95% CI: 0.3 to 9.63)). Conclusion: The results highlight the necessity of assessing function and concern regarding urinary catheter use and its impact on QoL, as well as its determinants. Evidence-based educational programs should be designed to enhance patients’ self-care abilities to relieve their sense of distress and enhance their confidence in caring for their catheters.

## 1. Introduction

An indwelling urinary catheter (IUC) is a common procedure in clinical practice that administered for patient’s safety. It is used for several reasons, including urinary incontinence, urinary retention, benign prostatic hyperplasia, consequences of traumatic injury, and other neurological diseases [1], where it drains the bladder [2]. Therefore, it is administered through (1) the urethral route where the catheter is inserted into the bladder through the urethra, or (2) the suprapubic route where the catheter is inserted into the bladder through a surgical incision above the pubis [3]. Urethral catheters (56%) are used more often than suprapubic catheters (44%) [4]. The duration of catheter use varies considerably, from 1 to 470 months [4].

The prevalence of urinary catheterization varies widely, depending on the setting (acute or critical care) and patient population [3,5]. Urinary catheter is often used to manage urinary retention and urinary incontinence or to facilitate monitoring of urine output [1]. The worldwide increase in the use of urological catheters is unsurprising, given recent figures from the World Health Organization [6], which estimated that 5% of the general population was affected by urinary incontinence, including 30% of the global elderly population and more than 50% of care home residents [6]. In the UK, more than 90,000 adults are estimated to have a urinary catheter, with 24% likely to develop catheter-associated urinary tract infection (CAUTI), which has adverse consequences including impaired quality of life (QoL), hospitalization, and increased mortality [7].

Factors contributing to a reduction in the prevalence and duration of urinary catheter use have been identified as good healthcare systems with adequate infrastructure and appropriately qualified urologists and nurses [8,9]. However, in developing nations, patients appear to be catheterized for a much longer period because of the lack of urologists, financial constraints to meet the cost of surgery, lack of equipment and expertise, and the greater presence of comorbidities [10,11]. Regular catheter changes in low-income countries are not always possible, and for ongoing catheter care, patients are often required to attend hospitals, usually with a close relative, which can be very burdensome.

Studies on the prevalence of long-term catheter use in Africa have been limited [12]. In Tanzania, a prevalence of 9.6% patients with urinary catheter living at home [12], which is higher than that of home care patients in the UK (5.4%) and the US (4.5%) [10,13].

A urinary catheter is associated with a range of complications and adverse events. It can cause stone formation, blockage, leakage, dislodgement, sediment, twists, bladder spasms, symptomatic bacterial infection, trauma, and hypersensitivity [2,4,14,15,16]. Catheterization can also cause other biopsychosocial complications including loss of dignity, loss of employment, sexual impairments, interruption in activity, social isolation, sexual restriction, and financial problems [17]. All of these factors have a serious impact on the quality of life and well-being of patients living with a long-term catheter [15,16,18].

The possible negative impact of a long-term indwelling urinary catheter use on patients’ QoL has been well-recognized [19,20]. This is partially due to the recurrent problems experienced by many patients, such as CAUTI, leaking, and catheter blockage [21,22]. In many areas of Africa, the risk of CAUTIs is estimated to be high, owing to unsterile environments, use of unsuitable catheter sizes, use of homemade drainage systems, and malnutrition [23]. However, the rates of CAUTI in developing countries are unknown because of the lack of surveillance data [24]. In Egypt, biofilm formation, largely caused by the bacterium *Klebsiella pneumoniae*, was the main cause of recurrent catheter-related infections (82.85%), especially among the elderly and those using the catheter for a long duration [25].

Despite the associated stigma and problems associated with catheter use, previous studies have described how patients come to accept it as necessary device [26]. Ensuring that the reasons for any problems, such as urinary catheter blocking or bypassing, are fully investigated by the nurse can help improve a patient’s quality of life (QoL) [27].

QoL is a multidimensional, complex concept that is known as an “individual’s perception of his/her position in life in the context of the culture and value systems in which they live and in relation to their goals, expectations, standards, and concerns” [28]. In this sense, exploring the QoL of patients with urinary catheters and the issues that affect their QoL, using a disease specific QoL questionnaire, are considered crucial to understanding their experiences and biopsychosocial needs.

Little is known about the patient perceptions of catheter functions and its impact on their QoL [19,29,30], especially in Egypt, where the resources are limited and burden of disease is high. There is growing recognition that assessing patients’ experience with urinary catheters and how they perceive its functions and its impact on their QoL is essential to improving their health outcomes and experience, and to relive barriers of caring. Therefore, this study is the first to use the Arabic version of a disease-specific QoL questionnaire to explore the views of patients living with a urinary catheter in Egypt on the urinary catheter functions and the catheter-related QoL.

### Research Questions

Two research questions were formulated to address this study’s aims.

1. Which specific areas are most affected when living with urinary catheters?
2. Which factors are significantly associated with impaired catheter-related QoL in patients with urinary catheter use?

## 2. Materials and Methods

The ‘Strengthening the Reporting of Observational Studies in Epidemiology’ (STROBE) checklist for cross-sectional studies was used to report this study.

### 2.1. Design and Setting

A cross-sectional study was conducted from April to September 2017 at urology wards and urology outpatient clinics in a University Hospital at Cairo City, the Capital of Egypt, where people from various socioeconomic backgrounds in Egypt attend to receive healthcare services.

### 2.2. Population, Criteria, and Sample Size

**Figure 1:**
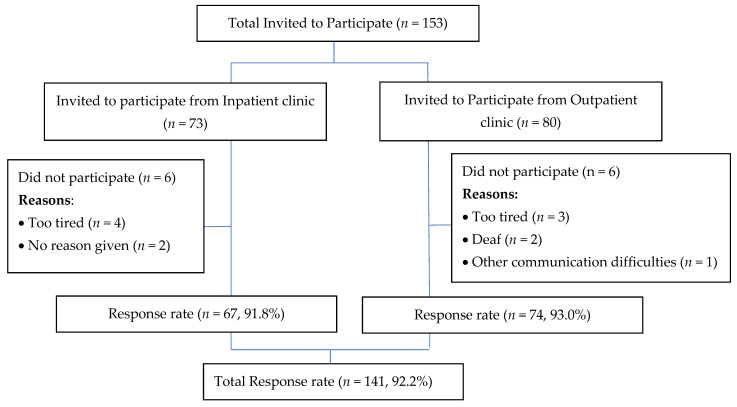
Recruitment flowchart.

The inclusion criteria for this study were patients aged ≥18 years living with a urinary catheter (suprapubic or urethral catheter) in place for ≥2 days and able to provide written consent to participate (Figure 1). The sample size for this study was calculated for psychometric validation of the Arabic version of the ICIQ-LTCQoL, as presented by Youssef et al. [31]. The analysis presented here is an exploratory secondary analysis.

### 2.3. Instruments

The International Consultation on Incontinence Questionnaire (ICIQ)-Long Term Catheter Quality of Life (LTCQoL) tool, developed by Cotterill et al. [29], provides a self-report evaluation in the specific area of indwelling catheter use. It consists of two scored domains: catheter function and concern (nine items; score range from 0–42; higher scores indicate worse QoL), lifestyle impact (three items; score range from 3–15; higher scores indicate worse QoL), and four stand-alone items relating to continence pads, pain, sexual activity, and bladder spasm. This psychometrically robust tool, which is easy to administer and takes less than 10 min to complete, provides a reliable and valid summary of the QoL of those living with a urinary catheter.

This questionnaire was successfully translated into Arabic, ensuring that it is a feasible and acceptable tool for measuring catheter-related QoL in Arabic-speaking individuals who use urinary catheters [31]. The Arabic version of the questionnaire showed satisfactory test–retest reliability. In addition, the Cronbach’s alpha was 0.75, indicating good internal consistency [31]. Permission to use the ICIQ-LTCQoL in this study was received from the copyright holder.

In addition to the Arabic ICIQ-LTCQoL, the participants were asked to complete a background datasheet that detailed sociodemographic data (i.e., gender, age, marital status, education, employment status, and type of work) and clinical variables (i.e., settings, disease duration, comorbidities, catheter duration, place of catheter, catheter material, and medical diagnosis).

### 2.4. Statistical Analysis

Statistical analyses were conducted using SPSS version 28. Data are described using (a) frequency and percentage distributions for categorical variables, (b) mean and standard deviation (*X* ± *SD*) for normally distributed continuous variables, and median and IQR for continuous data that were not normally distributed. Differences in QoL were explored using a series of univariate linear regressions. Three dependent variables were analyzed: total ICIQ-LTCQoL score and two subscales, catheter function and concern, and lifestyle impact. The explanatory variables included in the models were gender, care setting, marital status, education, employment status, type of catheter, catheter material, type of work, duration of catheter use, and age. The threshold for statistical significance was set to 0.05.

### 2.5. Ethical Considerations

Ethical approval was granted by the Research Ethics Committee of the Faculty of Nursing, Cairo University (approval code 2017-26). Official permission to access the settings for data collection was obtained from the head of the hospital, the head of the urology department, and outpatient clinics. Verbal and written information detailing the study aims and objectives were provided to all potential participants. The participants provided written informed consent prior to data collection. The confidentiality of participants was maintained throughout the study. All questionnaire data were coded to maintain participants’ confidentiality, and the data were saved on a secure computer that could be accessed by the researchers only. This study was conducted in accordance with the principles of the Declaration of Helsinki.

## 3. Results

### 3.1. Characteristics of the Participants

Of the 153 eligible patients invited to participate in this study, 141 were enrolled. A total of 110 (78.0%) were males with mean age of 53.8 years (SD: 16.4) (age ranged–18–86 years). A total of 110 participants (78.0%) were married, 81 (57.4%) had no formal education (uneducated), and 86 (61.0%) were employed (Table 1).

A total of 67 (47.5%) were inpatients and 74 (52.5%) were outpatients. The median duration of the medical condition since the diagnosis by the physician was 240 days (IQR: 60–750). The median duration of catheter use was 28 days (IQR: 15–90), and 75.2% had urethral catheter. The participants had various medical diagnoses, as shown in Table 2, which resulted in the use of a urinary catheter. A total of 34.0% of participants had comorbidities.

### 3.2. Catheter Impact on QoL

#### Catheter Function and Concern

**Figure 2:**
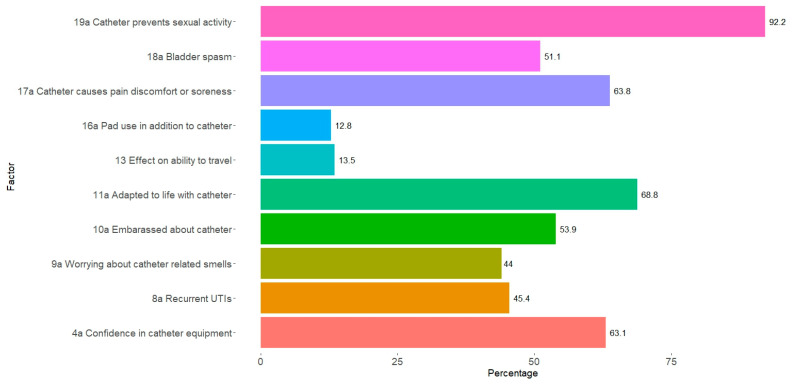
Prevalence of catheter-related problems affecting social activities and lifestyle as reported by participants (*n* = 141).

A total of 70.9% (*n* = 100) of the participants reported problems with the catheter function. The questions on which participants endorsed the highest levels of impact on QoL were embarrassment about their catheter (*n* = 76, 53.9%), experiencing urinary tract infections (UTIs) as a result of the catheter, causing them to feel unwell or requiring them to take antibiotics (*n* = 64, 45.4%), and lack of confidence in catheter equipment (*n* = 89, 63.1%). Over a quarter (27.7%, *n* = 39) stated that UTIs affected them “*several times per month*”. Worrying about catheter-related smells was reported by 44% (*n* = 62) of participants (Figure 2 shows the prevalence of catheter-related problems).

The factors over which participants reported the lowest levels of concern and stated that it was not an issue at all were ‘using pads to manage leaks’ (*n* = 123, 87.2%) and ‘catheter blockages’ (*n* = 81, 57.4%).

### 3.3. Catheter Impact on Lifestyle

Overall, 92.2% (*n* = 130) of the participants reported that having a catheter affected their daily lives. The negative impact of the catheter on both social activities and going out of the house was noted by 31.2% (*n* = 44) of the participants. The most commonly reported catheter-related problems were bladder spasm (*n* = 72, 51.1%); prevention of sexual activity (*n* = 130, 92.2%); and pain, discomfort, or soreness (*n* = 90, 63.8%). A majority, 68.8% (*n* = 97) felt that they had adapted to life with the catheter. However, only 12.8 (18%) stated that the catheter helped them leave the house, and only 42.6% (*n* = 60) reported that the catheter had no effect on their ability to go out of the house (Figure 2).

### 3.4. Factors Associated with QoL

Table 3 shows the results of fitting a series of univariate regression models to the ICIQ-LTCQoL results. Three variables were associated with catheter-related QoL: inpatient status, catheter material, and type of work. Whether the patient was an inpatient or outpatient was significantly associated with the total score (mean difference 6.34 (95% CI: 3.00 to 9.73)) and both subscales (catheter function subscale: mean difference = 4.92 (95% CI: 2.12 to 7.73), and lifestyle impact subscale: mean difference = 1.44 (95% CI: 0.25 to 2.63)). This finding indicates that outpatients had poorer catheter-related QoL than inpatients did.

Moreover, catheter material was significantly related to the catheter function domain of the ICIQ-LTCQoL, with Silicone Foley Catheter (100% Silicon) users experiencing poorer QoL related to catheter function than those with Latex Foley Catheter (Silicon-coated) (mean difference 4.43 (95% CI 0.62 to 8.24)). Workers/employees were found to have poorer catheter-related QoL than those who were retired (mean difference = 4.94 (95% CI 0.26 to 9.63)).

## 4. Discussion 

Urinary catheterization is a commonly used device for managing patients with lower urinary tract problems worldwide [12]. The duration of catheter use varies according to indications, from short-to long-term catheterization. A urinary catheter is considered short-term if it is in situ for less than 30 days and long-term if it is in situ for >30 days [32]. However, long-term catheterization has been noted to greatly impact patient QoL [19,33]. Limited literature exists on the number of people in Arabic-speaking nations who use urinary catheters. One of the few published papers highlighted that the prevalence of patients living at home with a long-term catheter in Tanzania was 9.6%, and that they were used for patients with two of the most common urological conditions in this population: benign prostatic hyperplasia and urethral strictures [12].

To the best of our knowledge, our study is the first to evaluate catheter-related QoL in patients with urinary catheters in Egypt. Our study examined areas that had the greatest impact on patients’ daily lives when living with a urinary catheter, and explored factors that were significantly associated with impaired QoL. Several studies have examined the experiences of patients with urinary catheters [19,30,34] and catheter-related factors that affect their daily lives [21,22]. However, more research is desperately required in developing countries, such as Egypt, to provide insights into catheter patients’ QoL and factors that may affect this using the LTCQoL. The LTCQoL was selected because it has two domains that assess catheter function, concern, and lifestyle impact, in addition to four items that assess the patient’s perception of pain, pads, bladder spasms, and sexual activity. The ICIQ-LTCQoL scale has been used in developed countries to measure the QoL of catheter users.

### 4.1. QoL of Patients with Urinary Catheters

Our study included 141 participants with a mean age of 53.8 years, who were almost equally recruited as outpatients and inpatients. Similar to other catheter studies, most of our participants were male [12,23,30]. Our results support a previous study showing that activities of daily living and QoL (using the WHO QoL Group questionnaire) in patients with a neurogenic bladder who underwent intermittent urinary catheterization were significantly affected [30].

Most participants reported that having a catheter affected their daily life. The categories of ‘Type of work’ in this study were ‘Housewife’, ‘Worker, ‘Retired, ‘Farmer’ and ‘Employee.’ Thus, for data analysis purposes, we grouped ‘Worker’ ‘farmer’ and ‘employee’ together as people who ‘work outside the home’. More than half of the participants recruited in this study were working outside the home and reported a lower catheter-related QoL than those who were retired. This is unsurprising, as many have reported how catheter-related problems affect their everyday lives, specifically their ability to go out of the house, perform social activities, and travel. Similarly, a previous qualitative study found that many factors shape the experience of patients with long-term catheters, such as going out of home, adjusting at night, catheter problems, social relations and interactions, support from surrounding people, unpredictability, intimacy relations, and body image [4,17,19].

In addition, a large proportion of our study participants reported that the frequency of urine infections, embarrassment from the catheter, lack of confidence in the catheter equipment, and concern about catheter-related smells had the greatest impacts on their QoL. The most commonly reported catheter-related problems were bladder spasms, prevention of sexual activity, pain, discomfort, and soreness. Previous studies have also shown that long-term catheter users experience similar catheter-related problems, such as leakage, blockage, encrustation, stone formation, painful bladder spasms, autonomic dysreflexia, and urethral trauma [13,17,33,35], which can affect their QoL, coping, independence, and daily activities [19,33].

It should be noted that more than half of our study participants felt that they had adapted to life with the catheter but only 18% stated that the catheter helped them go out of the house and that it had no effect on their ability to do this. A previous hermeneutic phenomenological study aimed at exploring and interpreting the lived experiences of patients with long-term catheters, revealed that, although the catheter was a part of their lives, they experienced stigma related to the visibility of the catheter [36]. Another qualitative study showed that participants demonstrated the ability to overcome catheter-related issues and could develop self-confidence, while others had struggled for longer to cope and live with the catheter [19]. Based on these findings, we suggest that providing individualized coping strategies on how patients manage their catheters and minimize urine accidents when out of their home can reduce the feeling of stigma related to catheter use. Further studies are required to establish the effectiveness of nursing management programs in improving patients’ QoL, self-care abilities, and coping strategies.

One question on which the largest proportion of participants expressed the highest level of impact on QoL was experiencing urine infections. These findings are important since they uncover one of the preventable problems (i.e., experiencing urine infections) that impairs the QoL of these populations. UTIs are among the most common hospital-associated infections [37], with 70–80% of UTIs attributed to the use of urinary catheters [38]. CAUTIs have been linked to increased morbidity, mortality, length of hospital stay, and costs [39]. Long-term catheterization has been found to be a significant factor associated with the increasing incidence of CAUTIs alongside other factors such as an unsterile environment during catheter insertion, poor quality of catheter material which is liable to encrustation, lack of urinary bags, catheter size, and the users’ health and nutritional condition [23]. It is therefore unsurprising that outpatient participants are more liable to catheter-related infections and other problems than hospitalized patients. Moreover, outpatient participants reported poorer QoL than inpatient participants in this study. This finding suggests that outpatient participants have insufficient supplies and knowledge to care for their catheter correctly or to control its associated problems. In a recent study, CAUTIs were significantly higher among outpatients [outpatients (82.2%) than among inpatients (35.3%) (*p* < 0.001)] [40]. Therefore, empowering patients with catheter-related knowledge, caring skills, and required supplies can decrease catheter-related complications, which can positively reflect on catheter-related QoL. Evidence-based guidelines that provide comprehensive recommendations for detecting and preventing CAUTIs must be applied while caring for catheter users, and healthcare providers must be educated on how to apply them.

### 4.2. Factors Impacting QoL of Patients with Urinary Catheters

Our study showed that catheter material was significantly related to the functional domain of the ICIQ-LTCQoL. The Latex Foley Catheter was the most commonly used catheter among our participants because it is less expensive than Silicone Foley Catheters. It is unexpected that patients with Latex Foley Catheters had better QoL than those with Silicone Foley Catheters. Catheter material has been found to contribute to several complications, such as UTIs [41]. There are several possible explanations for our findings. First, it could be that the patients with a Latex catheter had fewer complications, such as UTI or catheter-associated discomfort compared to those with Silicon catheters. Second, it is possible that the patients with Latex catheters had better communications with their healthcare providers, since it should be replaced every two weeks according to the policy, while the Silicon catheters must be replaced every 4 weeks. Third, it could be that the patients with Latex catheters had other factors unrelated to the catheter itself that contributed to their better QoL, such as better social support. Further research is needed to better understand the reasons for these unexpected findings and to explore the potential benefits and drawbacks of different types of Foley catheters on patients’ QoL.

In fact, evidence regarding catheter materials’ impact on patients’ QoL and activities of daily living is lacking. The Foley catheter has a lack of innovation in design where patients and healthcare providers suggested improving its design and practice [42]. Therefore, there is a need for modern innovative catheter design and materials that can improve the patients’ QoL [2,42].

On the other hand, it has been estimated that up to 60% of CAUTIs can be prevented by following evidence-based infection prevention guidelines [43]. A previous study indicated that nurses’ knowledge of catheter management could have a significant impact on addressing the problems of long-term catheter use and CAUTIs [42,44]. Early catheter removal has been recommended as the most effective preventive measure for reducing CAUTIs [45], and this is more likely to occur in a timely manner for inpatients, where health professionals are already caring for these individuals, compared to those being cared for at home.

In addition, a recent study found that older age, lower education, and catheter duration ≥ 6 weeks were predictors of CAUTIs among outpatients, whereas female sex and catheter bags not freely hanging were predictors of CAUTIs among inpatients [40]. This finding indicates the need for more education for outpatient participants regarding how to provide self-care for their catheters. A previous qualitative study explored the information needs of people living in a community with a long-term catheter (≥3 months) [22]. They found that patients with intermittent urinary catheters had a lack of information about catheter care and how to prevent related problems such as blockage, leaking, and infection. In addition, patients wanted information about managing the catheter’s impact on their sexual activity and social life [22]. Catheter users are more likely to experience poor QoL because of treatable factors, which, if considered in an interventional program, might improve patients’ QoL and help them cope with catheter-related issues.

## 5. Limitations and Recommendations

Our study has a number of limitations that must be considered in future research. Despite these limitations, our study provides valuable insights into the impact of catheter on QoL, and highlights the need for further research in this area. (1) The adoption of a cross-sectional design made it impossible to develop a causal link between the study variables and QoL. Therefore, a large-scale longitudinal study using the same questionnaire is required to develop causal relationships among the studied variables. (2) Recruiting participants from one hospital limits the generalizability of the results to other settings. However, these findings can be used to generate hypotheses for future research among these populations, as there is a lack of knowledge regarding catheter-related QoL and its predictors. (3) Due to patients’ vulnerability to tiredness due to their health condition, we were not able to include more variables that might affect QoL, such as the support they received from healthcare providers, family, and spouse, and their economic status. As a result, our study may not provide a comprehensive understanding of all predictors of QoL in patients who use urinary catheters. Thus, future research could build upon our findings by studying the relationship between catheter material and QoL using a qualitative or mixed methods design to provide a full picture of the relationship and explore other hidden factors. This would allow for a more comprehensive evaluation of the impact of catheter material on patients’ QoL, health outcomes, and clinical decisions.

Evidence-based educational programs should be designed to enhance patients’ self-care abilities to relieve their sense of distress and enhance their confidence in caring for their catheters. Further studies in the same population are needed to establish evidence of the impact of silicone catheters on patient QoL. Other factors that could explain the QoL and functional health status of these patients in Egypt are needed, such as their mental health status and coping strategies. Educating nurses on evidence-based catheter care and how to coach patients and their relatives is suggested, as it can improve their QoL and functional abilities. Further studies are needed to explore catheter-related problems and self-care abilities in patients with catheters, particularly in Egypt.

## 6. Conclusions

The results highlight the necessity of assessing function and concern regarding urinary catheter use and its impact on QoL, as well as its determinants. Urinary catheterization has a negative impact on patients’ QoL, especially in terms of work and social activities. In this study, workers, outpatients, and Silicone Foley Catheter materials were found to be independent factors that were significantly associated with impaired QoL. This finding is particularly important because it could enhance the insight of healthcare providers regarding the main factors that require further attention while caring for or following up patients with urinary catheters. Healthcare professionals, particularly nurses, should continually assess catheter-related complications and teach patients how to recognize their related signs and symptoms, as well as self-care for related issues.

## Figures and Tables

**Table 1:** Demographic characteristics of the participants, *n* = 141.

Variable		N (%)
Age	Mean ± SD	53.8 ± 16.4
	Median	57 years
Gender	Female	31 (21.99)
	Male	110 (78.01)
Marital status	Unmarried	31 (21.99)
	Married	110 (78.01)
Education	Uneducated	81 (57.45)
	Primary/preparatory	26 (18.44)
	Secondary	24 (17.02)
	Higher education	10 (7.09)
Current employment status	Employed	86 (60.99)
	Unemployed (i.e., sick leave/housewife/retired)	55 (39.01)

**Table 2:** Clinical variables of the participants, *n* = 141.

Variable		N (%)
Settings	Inpatient	67 (47.50)
	Outpatient	74 (52.50)
Disease duration (in days)	Mean ± SD	831.01 ± 1628.59
	Median (interquartile range)	240 (60–750)
Have comorbidity	No	93 (66.00)
	Yes	48 (34.00)
Number of comorbidities	1	31 (64.58)
	2	15 (31.25)
	3	2 (4.17)
Catheter duration (in days)	Median (interquartile range)	28 (15–90)
	Range	2–9125
Place of catheter	Urethral	106 (75.20)
	Supra-pubic	35 (24.80)
Catheter material	Silicon Foley Catheter (100% Silicon)	24 (17.00)
	Latex Foley Catheter (coated with Silicon)	117 (83.00)
Medical diagnosis (Reason for urinary catheter use)	Bladder disorders (cancer, hole, mass, rupture, problems, cystectomy, test injury)	28 (19.86)
	Bleeding or urine retention	11 (7.80)
	Prostate disorders (enlargement, inflammation, prostatectomy)	29 (20.57)
	Fistula after hypospadias, urethra fistula, hole in the urinary pathway, obstruction of urinary pathway	15 (10.64)
	Hysterectomy	1 (0.71)
	Stones	27 (19.15)
	Long-segment urethral stricture	1 (0.71)
	Pelvic fracture, pelvic mass, fracture	4 (2.84)
	Post ileocytoplast	1 (0.71)
	Renal impairment	10 (7.09)
	Spinal cord injury, stroke, Paraplegia	6 (4.26)
	Ureter cancer, mass, injury, ureterovesical reflux	5 (3.55)
	Urethral rupture, stenosis, injury	3 (2.13)

**Table 3:** Univariate regression of ICIQ-LTCQoL and its subscales (*n* = 141).

Variable	Value	Mean Total ICIQ-LTCQoL Score	Unadjusted Regression Coefficient-Total ICIQ-LTCQoL	Mean Function Score	Unadjusted Regression Coefficient-Catheter Function and Concern Subscale	Mean Lifestyle Score	Unadjusted Regression Coefficient-Lifestyle Impact Subscale
Gender	Male	30.01	3.65 (−0.55 to 7.86)	19.88	2.91 (−0.57 to 6.40)	10.13	0.74 (−0.72 to 2.20)
	Female	26.35	ref	16.97	ref	9.39	ref
Setting	Inpatient	25.87	ref	16.66	ref	9.21	ref
	Outpatient	32.23	6.34 (3.00 to 9.73) **	21.58	4.92 (2.12 to 7.73) **	10.65	1.44 (0.25 to 2.63) **
Marital status	Married	28.76	ref	18.65	ref	10.11	ref
	Unmarried	30.77	2.01 (−2.23 to 6.25)	21.32	2.67 (−0.83 to 6.16)	9.45	−0.66 (−2.11 to 0.80)
Education	No education	28.91	0.71 (−6.34 to 7.76)	18.89	0.59 (−5.25 to 6.42)	10.02	0.13 (−2.30 to 2.55)
	Primary/preparatory	29.35	1.15 (−6.68 to 8.97)	19.81	1.51 (−4.97 to 7.98)	9.54	−0.36 (−3.05 to 2.33)
	Secondary	30.46	2.26 (−5.66 to 10.17)	20.21	1.91 (−4.97 to 7.98)	10.25	0.35 (−2.37 to 3.07)
	Higher education	28.20	ref	18.30	ref	9.90	ref
Employment status	Employed	30.82	ref	20.55	ref	10.27	ref
	Not employed	28.17	−2.64 (−6.23 to 0.94)	18.41	−2.14 (−5.11 to 0.83)	9.77	−0.51 (−1.74 to 0.73)
Type of catheter	Suprapubic	29.97	1.01 (−3.06 to 5.09)	19.97	0.97 (−2.40 to 4.34)	10.00	0.05 (−1.35 to 1.45)
	Urethral	28.95	ref	19.00	ref	9.95	ref
Catheter material	Silicone Foley Catheter	32.96	4.52 (−0.10 to 9.15)	22.92	4.43 (0.62 to 8.24) **	10.04	0.09 (−1.52 to 1.70)
	Latex Foley Catheter	28.44	ref	18.49	ref	9.95	
Type of work	Housewife	25.62	−0.63 (−6.25 to 4.99)	16.66	0.74 (−3.89 to 5.37)	8.97	−1.37 (−3.34 to 0.60)
	Work outside the home (i.e., Worker/employee/farmer)	31.19	4.94 (0.26 to 9.63) **	21.00	5.08 (1.22 to 8.95) **	10.19	−0.14 (−1.78 to 1.50)
	Retired	26.25	ref	15.92	ref	10.33	ref
Catheter duration	One month or less	28.13	−2.29 (−5.80 to 1.22)	18.04	−2.57 (−5.46 to 0.33)	10.09	0.28 (−0.94 to 1.49)
	Over one month	30.42	ref	20.61	ref	9.82	ref
Age	Continuous	-	−0.10 (−0.21 to 0.001)	-	−0.09 (−0.18 to −0.003) **	-	−0.01 (−0.05 to 0.03)

** *p* < 0.05 is statistically significant.

## Data Availability

Data can be obtained from the corresponding author upon reasonable request.
